# Effects of propofol versus sevoflurane induction on echocardiographic parameters in patients with mitral stenosis: a randomized clinical trial

**DOI:** 10.1016/j.bjane.2026.844772

**Published:** 2026-06-03

**Authors:** Mohammed Jaffer Sherif, Saravana Babu MS, Prasanta Kumar Dash, Nagarjuna Panidappu, Subin Sukesan, Thomas Koshy

**Affiliations:** a Sree Chitra Tirunal Institute for Medical Sciences and Technology, Division of Cardiothoracic and Vascular Anesthesia, Trivandrum, India; b All India Institute of Medical Sciences, Department of Anesthesiology, Madurai, India; c Amrita Institute of Medical Sciences, Department of Cardiothoracic and Vascular Anesthesia, Kochi, India

**Keywords:** Anesthetics, Inhalation, Anesthetics, Intravenous, Echocardiography, Mitral valve stenosis, Propofol, Sevoflurane

## Abstract

**Background:**

The main aim of this study was to compare the effects of propofol and sevoflurane induction on the echocardiographic parameters of Mitral Stenosis (MS) patients.

**Methods:**

Prospective, randomized, outcome-assessor-blinded clinical trial in 80 adults with MS undergoing mitral valve replacement. Patients were randomized to receive General Anesthesia (GA) induction with either propofol (Group P) or sevoflurane (Group S). The primary objective was to assess the changes in mean gradient after 3 minutes of GA induction. Secondary objectives were to assess the changes in echocardiographic Doppler parameters and hemodynamic variables.

**Results:**

Compared to pre-induction values, there was significant reduction in the peak and mean gradients as well as peak velocity, in both the groups after GA induction. The Mitral Valve Area calculated by continuity equation (MVA-c) and by pressure half time (MVA-p) were increased in Group S after GA induction. On comparison between the two groups, Group S showed lower pressure half time and greater MVA-p (p = 0.0099) and MVA-c (p = 0.0316) than Group P after GA induction. Mean arterial pressure decreased at the 1^st^ and 3^rd^ minute following GA induction in both groups. The Heart Rate (HR) increased at the 1^st^ and 3^rd^ minute in Group P whereas it reduced at both the time points in group S.

**Conclusion:**

Even though both anesthetic agents reduced transvalvular gradients in MS patients, sevoflurane was associated with a greater increase in estimated valve area and a reduction in heart rate compared with propofol. These findings suggest more favorable short-term hemodynamic and echocardiographic changes with sevoflurane at the studied time points.

Mitral Stenosis (MS) is one of the most common valvular heart diseases and the major manifestation of rheumatic fever, the incidence of which is declining in developed countries.^1^ Echocardiographic modalities such as M-mode, two-dimensional echo, three-dimensional echo, and Doppler evaluation are utilized for assessment of the severity of the lesion.^2^ The echocardiographic parameters determining the severity of mitral valve stenosis are greatly influenced by the hemodynamic status of the patient.^3^ Patients with MS are subjected to General Anesthesia (GA) for various reasons like transesophageal echocardiographic evaluation, percutaneous balloon mitral valvotomy, and elective or emergency Mitral Valve Replacement (MVR).^4^ A clear understanding of cardiovascular dynamics in Mitral Stenosis (MS) is essential when selecting an anesthetic regimen for general anesthesia induction. Progressive narrowing of the mitral valve impairs left ventricular filling, elevating left atrial pressure and leading to atrial dilatation; although disease severity is defined by valve area, symptoms are primarily driven by left atrial pressure. Factors that increase transvalvular gradients, such as tachycardia or increased cardiac output, can precipitate pulmonary congestion and edema.^5^^,^^6^ Chronic left ventricular underfilling, compounded by interventricular septal shift from right ventricular hypertrophy, may result in systolic dysfunction.^7^ Because cardiac output in MS is fixed and preload dependent, induction of anesthesia is associated with significant hemodynamic instability. Therefore, anesthetic goals include avoiding tachycardia and preserving preload, afterload, and left ventricular contractility. Propofol and sevoflurane are commonly used agents for GA induction; however, their effects on the hemodynamic and echocardiographic profiles of patients with MS have not been extensively studied. Although sevoflurane is considered relatively more cardiostable than propofol, the extent of its influence on flow dynamics in MS and its clinical significance remain poorly understood.

We hypothesize that sevoflurane, compared with propofol, may be associated with more favorable hemodynamic parameters after GA induction in patients with MS, owing to its potential to reduce heart rate, which may in turn improve valvular flow dynamics. Therefore, we designed a prospective, randomized, comparative study to evaluate and compare the effects of propofol- and sevoflurane-based induction on echocardiographic parameters in patients with MS undergoing MVR. The primary objective of the study was to assess and compare the change in mean transmitral pressure gradient three minutes after induction of GA. Changes in other echocardiographic and Doppler parameters, as well as hemodynamic variables, were considered secondary objectives of the study.

## Methods

This prospective, randomized, outcome-assessor-blinded single center clinical trial was conducted in accordance with the Declaration of Helsinki, with approval from the institutional ethics committee (SCT/IEC/1509/JAN-2023) and following prospective trial registration with the clinical trials registry of India (Registration Number: CTRI/2023/08/056224; Date: 07, August 2023, study period 10-Aug-2023 to 30-Dec-2023). Written informed consent was obtained from all the study participants and the study adhered to the Consolidated Standards of Reporting Trials (CONSORT) guidelines for reporting parallel groups.^8^ Provisionally accepted patients were screened for eligibility to participate in the study. Patients aged more than 18 years of either sex, who were diagnosed with MS and scheduled to undergo elective MVR were enrolled sequentially into the study. Exclusion criteria were patients not willing to participate in the study, more than mild mitral regurgitation, associated aortic, pulmonary, and tricuspid valve disease, associated symptomatic coronary artery disease, on preoperative inotropic or mechanical circulatory supports, redo surgeries and emergency surgeries. We also planned to exclude patients with suboptimal pre-induction or post-induction Transthoracic Echocardiographic (TTE) images.

After screening and enrolling the patients for the study, all study participants were randomly allocated to one of the study groups based on a computer-generated random number table. The type of intervention the subjects were about to receive on the day of surgery was concealed in an opaque sealed envelope. Those envelopes were handed over to that particular anesthesiologist attending the case. The study participants received either propofol (Group P) or sevoflurane (Group S) for induction of GA based on randomization and group allocation. An independent echocardiographer performed the TTE assessment as per the study protocol. The hemodynamic and echocardiographic data were recorded and analyzed by an outcome assessor.

Patients included in the study were educated about the study during preoperative evaluation. In the operating room, after attaching the ASA standard monitors, invasive arterial cannulation was done under local anesthesia for all the patients. Additionally, Bi-Spectral Index (BIS; Medtronic, Minneapolis, MN, USA) sensors were placed on the forehead to monitor the depth of anesthesia. Induction of general anesthesia was performed using fentanyl (5 µg.kg^-1^) and midazolam (0.05 mg.kg^-1^) in both groups. In Group S, anesthesia was induced with sevoflurane, starting at 2% and gradually increasing to 6% in 100% oxygen, with patients breathing spontaneously until loss of the eyelash reflex. In Group P, induction was performed with propofol at 1 mg.kg^-1^, followed by 10 mg boluses until loss of the eyelash reflex. In both groups, anesthetic depth was titrated to maintain a Bispectral Index (BIS) between 40 and 60. This was followed by administration of vecuronium 0.1 mg.kg^-1^ to facilitate tracheal intubation. Titrated boluses of 25 μg of injection phenylephrine was administered when the Mean Arterial Pressure (MAP) reduced more than 20% from the baseline. Anesthesia was maintained with sevoflurane, fentanyl infusion, and intermittent boluses of vecuronium.

**Figure 1:**
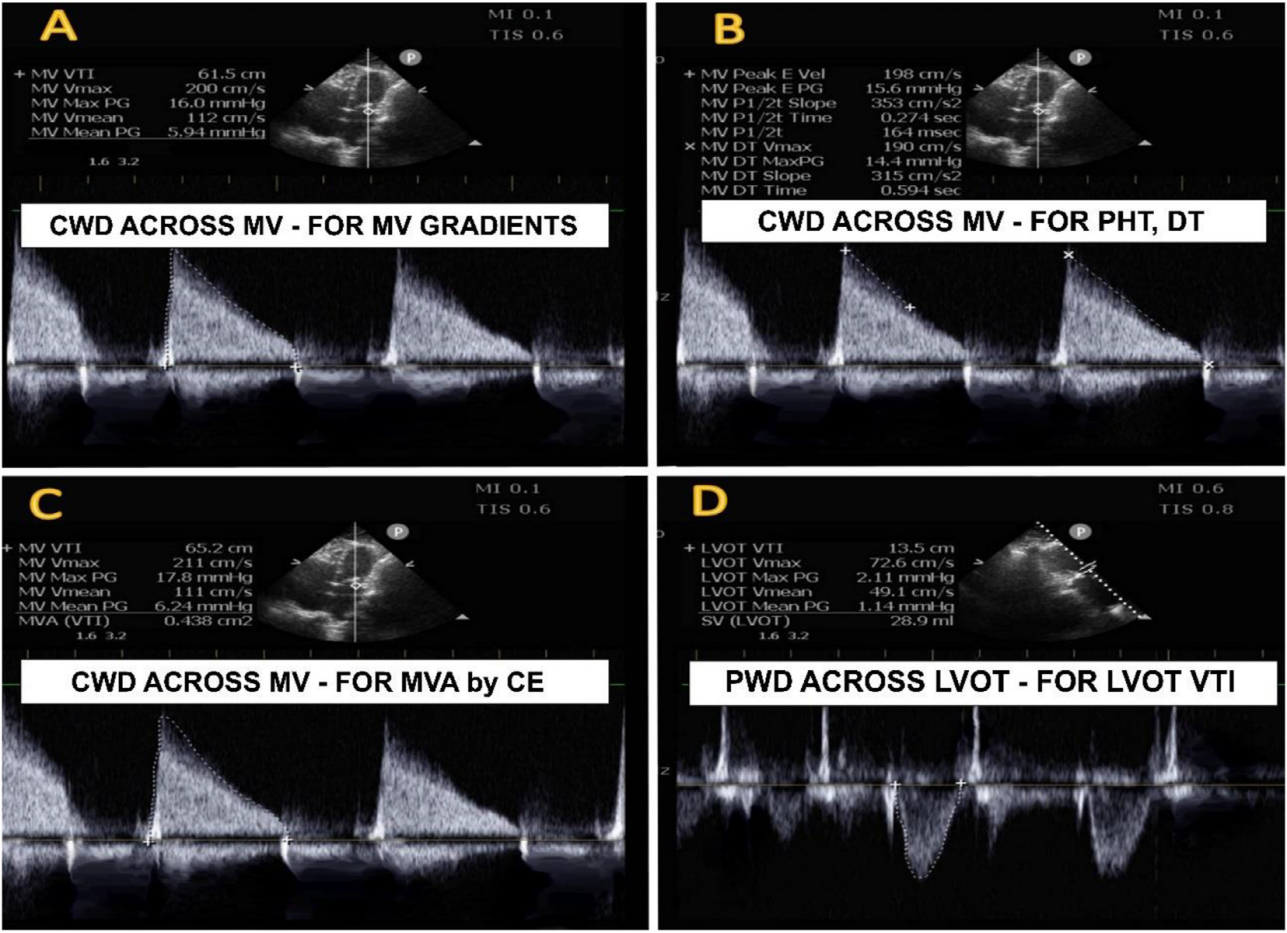
Images showing various echocardiographic parameters to determine the severity of MS. (A) CWD across mitral valve in transthoracic apical four-chamber view-spectral Doppler traced to determine the Vmax-maximum velocity, peak, and mean pressure gradients. (B) CWD across mitral valve in transthoracic apical four-chamber view-spectral Doppler traced along the slope starting from the peak to half the velocity for determination of PHT and till the baseline to derive DT. (C) CWD across mitral valve in transthoracic apical four-chamber view-spectral Doppler traced to obtain MV VTI for the derivation of MVA by continuity equation. (D) LVOT VTI was obtained by tracing the PWD spectral trace across LVOT in an apical five-chamber view. CE, Continuity Equation; CWD, Continuous Wave Doppler; DT, Deceleration Time; LVOT, Left Ventricular Outflow Tract; MV, Mitral Valve; MVA, Mitral Valve Area; PHT, Pressure Half Time; PLAX, Parasternal Long Axis view; PWD, Pulse Wave Doppler; VTI, Velocity Time Integral.

Before GA induction (T-0) and after 3 minutes of GA induction, but before tracheal intubation (T-1), TTE examination of the heart was performed by an experienced perioperative echocardiographer using an ultrasound machine (S5-1; iE33; Philips, Bothell, USA) and the images were stored for analysis (Fig. 1). An independent outcome assessor blinded to the type of intervention analyzed the following parameters from the stored echocardiographic data: 1) Peak Velocity (PV), 2) Peak Pressure Gradient (PPG), 3) Mean Pressure Gradient (MPG), 4) Pressure Half Time (PHT), 5) Deceleration Time (DT), 6) Mitral Valve Area by continuity equation (MVA-c) and 7) Mitral Valve Area by PHT (MVA-p). Parameters 1 to 5 were derived by tracing the transmitral continuous wave spectral Doppler trace in the apical 4 chamber view (Fig. 1A‒B). MVA-c was determined from Velocity Time Integral (VTI) of the mitral valve (Fig. 1C), VTI of Left Ventricular Outflow Tract (LVOT) (Fig. 1D), and LVOT diameter [MVA-c = (LVOT area × LVOT VTI) / mitral valve VTI].^9^^,^^10^ The MVA-p is determined by the formula: MVA-p = (220/PHT).^6^ All the echocardiographic assessments were performed according to the latest American Society of Echocardiography (ASE) guidelines by averaging 3 to 5 cardiac cycles in patients with sinus rhythm and 7 to 10 cardiac cycles in patients with atrial fibrillation.^11^

The patient's hemodynamic parameters like MAP, Heart Rate (HR), and Cardiac Output (CO) were noted before induction (baseline), at 1-minute, and at 3 minutes after induction of GA. The total dose of phenylephrine required during induction to restore the MAP close to the baseline was recorded. To test the reproducibility of Doppler measurements 10 sets of data were collected randomly. The data were analyzed by a single assessor at two different time points for the intra-observer variability and by an additional assessor for interobserver variability.

Based on a pilot study involving 10 patients that demonstrated an effect size of 0.5 standard deviations (Cohen’s *d* = 0.5), a sample size of 36 participants per group was calculated to be sufficient to detect a 25% change in MPG between pre- and post-induction values within each group, with 80% statistical power and a significance level (α) of 0.05. We included 40 patients in each group considering a 10% drop-out rate due to technical reasons like poor quality echocardiographic images. All statistical analyses were performed using the statistical software package SPSS version 23.0 (IBM Software Group, Chicago, IL 60606, USA). Categorical and continuous variables were expressed as number (proportions) and mean ± SD or median (Interquartile Range; IQR; 25^th^−75^th^ percentiles) respectively. The Kolmogorov-Smirnov test was used to test the normality of the data. A paired *t*-test was used to compare quantitative parameters before and after intervention in each group. An independent *t*-test was used to compare quantitative parameters between the two groups. Pearson Chi-Square test was used for the analysis of categorical variables. The intra-class correlation coefficient was calculated for intra and inter-observer reliability assessment. An intra-class correlation coefficient of 0.7 to 1 is considered to be a good correlation. For all statistical interpretations, a p-value of < 0.05 was considered to be statistically significant.

## Results

**Figure 2:**
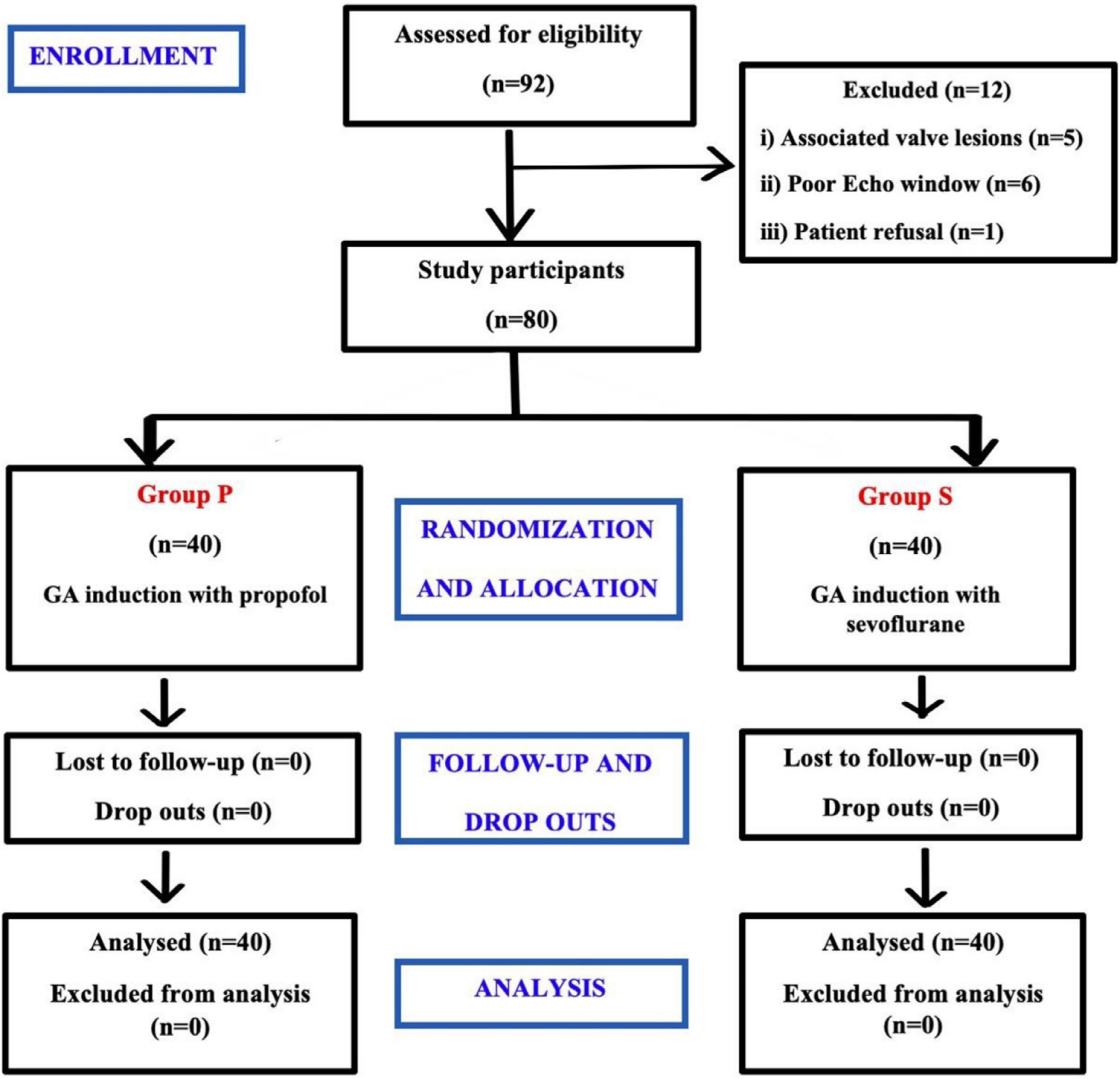
CONSORT diagram depicting various stages at which patients were screened and finally recruited into the study.

A total of 92 patients diagnosed with MS and scheduled for elective MVR were assessed for eligibility. Of these, 12 patients were excluded based on predefined exclusion criteria. The remaining 80 patients were randomly allocated into two groups, with 40 patients in each group. No patients were excluded after randomization because of suboptimal pre- or post-induction TTE images and all randomized participants were included in the final analysis (Fig. 2). Both groups were comparable for demography, functional class, and preoperative echocardiographic data (Table 1). In Group P, after the induction of GA, there was a significant fall in MPG and PV when compared to the baseline pre-induction values (Table 2). In Group S, besides a significant decrease in post-induction MPG, PPG and PV, there was also a significant decrease in PHT and DT (Table 2). This significant decrease in PHT was reflected as an increase in post-induction MVA-p in comparison to pre-induction MVA-p in Group S (Table 2). The MVA-c also showed a significant increase in the post-induction period in comparison to pre-induction values in Group S (Table 2). The pre-induction and post-induction values of PHT, DT, MVA-c, and MVA-p did not show any significant change in Group P (Table 2).

**Table 1:** Comparison of demographic data, functional class, and preoperative echocardiographic parameters.

Variables		Group P (n = 40)	Group S (n = 40)	p-value
Age (years)		50 ± 7.0	49.6 ± 7.2	0.8150^a^
Weight (kg)		62.5 ± 6.8	66.2 ± 10	0.0540^a^
Gender	Male	8 (20%)	16 (40.0%)	0.0510^b^
	Female	32 (80%)	24 (60%)	
NYHA class	II	20 (50.0%)	22 (55.0%)	0.654^b^
	III	20 (50.0%)	18 (45.0%)	
Heart rhythm	Sinus	24 (60.0%)	24 (60.0%)	1.0000^b^
	Non- Sinus	16 (40.0%)	16 (40.0%)	
Preoperative TTE	MPG (mmHg)	12.09 ± 4.12	13.18 ± 5.10	0.2963^a^
	MVA (cm^2)^	0.93 ± 0.20	0.96 ± 0.24	0.5454^a^
Pre-induction TTE	MPG (mmHg)	11.12 ± 3.02	11.30 ± 4.84	0.8424^a^
	MVA (cm^2^)	0.88 ± 0.21	0.91 ± 0.11	0.4259^a^

MAP, Mean Arterial Pressure; MPG, Mean Pressure Gradient; MVA, Mitral Valve Area; NYHA, New York Heart Association; TTE, Transthoracic Echocardiography.

a Using *t*-test, data expressed as mean and Standard Deviation (SD).

b Using Chi-Square test, data expressed as frequency.

**Table 2:** Comparison of pre-induction and post-induction echocardiographic and Doppler parameters.

	Group P (n = 40)				Group S (n = 40)			
Doppler parameters	Pre-induction	Post-induction	Δ (95% CI)	p-value	Pre-induction	Post-induction	Δ (95% CI)	p-value
PV (m.s^-1^)	2.2 ± 0.3	1.8 ± 0.4	0.4 (0.29 to 0.52)	< 0.0001	2.3 ± 0.4	1.9 ± 0.3	0.4 (0.29 to 0.52)	< 0.0001^a^
PPG (mmHg)	20.4 ± 6.1	13.9 ± 5.9	6.5 (4.58 to 8.42)	< 0.0001	20.4 ± 6.0	14.6 ± 4.0	5.8 (4.10 to 7.50)	< 0.0001^a^
MPG (mmHg)	11.1 ± 3.0	7.1 ± 2.8	4 (3.07 to 4.93)	< 0.0001	11.3 ± 4.8	8.0 ± 3.1	3.3 (1.95 to 4.65)	< 0.0001^a^
PHT (msec)	261.9 ± 66.9	250.3 ± 55.8	11.6 (-8.2 to 31.4)	0.4023	240.6 ± 57.5	224.6 ± 58.5	16 (2.5 to 34.5)	< 0.0001^a^
DT (msec)	846.0 ± 192.8	831.4 ± 250.8	14.6 (-58.0 to 87.2)	0.7711	811.8 ± 201.7	767.6 ± 179.7	44.2 (17 to 105.4)	0.0030^a^
MVA-c (cm^2^)	0.90 ± 0.21	0.91 ± 0.26	0.01 (-0.066 to 0.086)	0.8504	0.99 ± 0.28	1.05 ± 0.31	0.06 (0.035 to 0.155)	0.0080^a^
MVA-p (cm^2^)	0.88 ± 0.21	0.92 ± 0.29	0.04 (-0.043 to 0.123	0.4819	0.91 ± 0.11	1.08 ± 0.25	0.17 (0.101 to 0.239)	0.0002^a^
Post induction MPG < 5 mmHg	12 (30%)				6 (15%)			0.1104^b^

CI, Confidence Interval; DT, Deceleration Time; MPG, Mean Pressure Gradient; MVA-c, Mitral Valve Area by continuity equation; MVA-p, Mitral Valve Area by PHT; PHT, Pressure Half Time; PPG, Peak Pressure Gradient; PV, Peak Velocity. Δ = Pre-induction value-post induction value.

a Using paired *t* test, Values are expressed as mean ± Standard Deviation.

b Using Chi-Square test, data expressed as frequency.

On comparison of post-induction echocardiographic parameters between the two groups, the PHT was significantly lower in Group S than in Group P whereas the MVA-p and MVA-c were significantly higher in Group S than in Group P (Table 3). The other post-induction echocardiographic parameters (PV, PPG, MPG, and DT) were comparable between the two groups. Baseline hemodynamic parameters (HR, MAP, and CO) were comparable between the two groups. The post-induction HR was significantly lower in Group S in the 1^st^ minute and 3^rd^ minute in comparison to Group P (Table 3). In both groups, the post-induction MAP and CO values decreased from the baseline and were comparable between the two groups (Table 3). The phenylephrine requirement to normalize the MAP was significantly higher in Group P (83%) in comparison to Group S 64% (50 ± 12.5 vs. 37.5 ± 12.5 μg, median [IQR]- 50 [25] vs. 37.5 [25], p < 0.0001). Intra-class correlation coefficient analysis of data (PV, PPG, MPG, PHT, and MVA-p) measured by different observers (inter-observer variability) and by the same observer at two different time points (intra-observer variability) showed statistically significant good correlation (Table 4).

**Table 3:** Comparison of post-induction echocardiographic, Doppler and hemodynamic parameters.

Parameters	Group P (n = 40)	Group S (n = 40)	Estimated mean difference (95% CI)	p-value
PV (m.s^-1^)	1.8 ± 0.4	1.9 ± 0.3	-0.1 (-0.257 to 0.0574)	0.2097^a^
PPG (mmHg)	13.9 ± 5.9	14.6 ± 4.0	-0.7 (-2.944 to 1.544)	0.5364^a^
MPG (mmHg)	7.1 ± 2.8	8.0 ± 3.1	-0.9 (-2.215 to 0.415)	0.1769^a^
PHT (msec)	250.3 ± 55.8	224.6 ± 58.5	25.7 (0.252 to 51.148)	0.0478^a^
DT (msec)	831.4 ± 250.8	767.6 ± 179.7	63.8 (-33.32 to 160.92)	0.1948^a^
MVA-c (cm^2^)	0.91 ± 0.26	1.05 ± 0.31	-0.14 (-0.267 to 0.0126)	0.0316^a^
MVA-p (cm^2^)	0.92 ± 0.29	1.08 ± 0.25	-0.16 (-0.281 to -0.0395)	0.0099^a^
HR (Beats.min^-1^) Baseline	83.8 ± 15.1	82.4 ± 19.5	1.4 (-6.363 to 9.163)	0.7210^a^^,^^b^
HR (Beats.min^-1^) 1^st^ minute	86.2 ± 14.8 (Δ = 2.4, 95% CI -2.37 to 7.17)	78.1 ± 18.1 (Δ = 4.3, 95% CI -1.72 to 10.32)	8.1 (0.74 to 15.46)	0.0310^a^^,^^b^
HR (Beats.min-^1^) 3^rd^ minute	89.1 ± 16.1 (Δ = 5.3, 95% CI 0.31 to 10.29)	76.7 ± 16.9 (Δ = 5.7, 95% CI -0.18 to 11.58)	12.4 (5.053 to 19.747)	0.0010^a^^,^^b^
MAP (mmHg) Baseline	105.0 ± 7.3	105.7 ± 10.5	-0.7 (-4.726 to 3.326)	0.7131^a^^,^^b^
MAP (mmHg) 1^st^ minute	91.6 ± 6.5 (Δ = 13.4, 95% CI 11.18 to 15.62)	89.1 ± 8.1 (Δ = 16.6, 95% CI 13.55 to 19.65)	2.5 (-0.769 to 5.769)	0.1263^a^^,^^b^
MAP (mmHg) 3^rd^ minute	85.8 ± 6.4 (Δ = 19.2, 95% CI 17.0 to 21.4)	85.4 ± 8.8 (Δ = 20.3, 95% CI 17.17 to 23.43)	0.4 (-3.025 to 3.825)	0.8173^a^^,^^b^
CO (L.min^-1^) Baseline	4.1 ± 0.8	4.0 ± 0.6	0.1 (-0.215 to 0.415)	0.5289^a^^,^^b^
CO (L.min^-1^) post-induction	3.1 ± 0.8 (Δ = 1, 95% CI 0.74 to 1.26)	2.9 ± 0.5 (Δ = 1.1, 95% CI 0.92 to 1.280)	0.2 (-0.097 to 0.497)	0.1839^a^^,^^b^

CI, Confidence Interval; CO, Cardiac Output, DT, Deceleration Time; HR, Heart Rate; MAP, Mean Arterial Pressure; MPG, Mean Pressure Gradient; MVA-c, Mitral Valve Area by continuity equation; MVA-p, Mitral Valve Area by PHT; PHT, Pressure Half Time; PPG, Peak Pressure Gradient; PV, Peak Velocity.

Estimated mean difference = mean ± standard deviation (Group P– Group S).

Δ = Baseline value – value at that time point (within the same group).

a Using *t*-test, data expressed as mean and Standard Deviation (SD).

b p-values (unadjusted) were obtained from separate pairwise comparisons and do not account for within-subject correlation (no repeated-measures model) and were not adjusted for multiple comparisons; therefore, results ‒ especially marginal p-values ‒ should be interpreted cautiously.

**Table 4:** Intra-class correlation coefficient analysis for inter and intra-observer reliability of data.

Intraclass correlation coefficient analysis								
	Inter-observer coefficient	95% CI		p-value	Intra-observer coefficient	95% CI		p-value
		Lower Bound	Upper Bound			Lower Bound	Upper Bound	
PV	0.994	0.976	0.999	< 0.0001	0.995	0.980	0.999	< 0.0001
PPG	0.992	0.968	0.998	< 0.0001	0.990	0.960	0.998	< 0.0001
MPG	0.991	0.965	0.998	< 0.0001	0.997	0.988	0.999	< 0.0001
PHT	0.992	0.970	0.998	< 0.0001	0.998	0.992	1.000	< 0.0001
MVA-p	0.986	0.942	0.996	< 0.0001	0.984	0.937	0.996	< 0.0001

CI, Confidence Interval; MPG, Mean Pressure Gradient; MVA-c, Mitral Valve Area by continuity equation; MVA-p, Mitral Valve Area by PHT; PHT, Pressure Half Time; PPG, Peak Pressure Gradient; PV, Peak Velocity. Intra-class correlation coefficient of 0.7 to 1 is a good correlation.

## Discussion

In this study, both propofol and sevoflurane were associated with reductions in MPG and PPG following induction of GA. A greater increase in estimated valve area was observed in the sevoflurane group. Hemodynamic changes were more pronounced with propofol, whereas sevoflurane was associated with more favorable hemodynamic effects. Under normal conditions, the flow velocity across the mitral valve is less than 1.3 m.s^-1^.^9^ As mitral valve area decreases with disease progression, transvalvular flow velocity increases. Induction of general anesthesia with either propofol or sevoflurane reduces sympathetic and parasympathetic tone and increases venous capacitance, leading to a reduction in venous return.^12^^,^^13^ The resulting decrease in blood flow across the mitral valve accounts for the observed reductions in peak velocity, peak pressure gradient, and mean pressure gradient. However, the updated guidelines on valvular heart diseases did not consider either velocity or pressure gradients for the classification of disease severity.^4^ This is because the trans-mitral Doppler is heavily influenced by the flow across the valve, which in turn is determined by the diastolic filling time and compliance of the left atrium and left ventricle.^14^^,^^15^ Despite these limitations, the MPG serves as a very important and convenient parameter to decide upon the severity of stenosis and has been in use for a longer time.^16^ Transmitral MPG of less than 5 mmHg indicates mild or no stenosis whereas MPG of more than 10 mmHg indicates severe stenosis.^17^^,^^18^ When this criterion was considered, it was surprising to find that approximately 23% of the total study patients in our study, who were documented to have severe MS, had their MPG less than 5 mmHg after induction of GA (Group P: 12 [30%] and Group S: 6 [15%]). This finding was predominantly seen in patients induced with propofol. This is a clinically alarming phenomenon under anesthesia, where misclassification of MS severity could easily happen.

The PHT is the next important echocardiographic parameter that indicates the severity of MS. According to the American College of Cardiology (ACC)/American Heart Association (AHA) 2020 guidelines, a mitral valve PHT of ≥ 150 msec is considered severe MS.^4^ All patients enrolled in our study had a baseline PHT of more than 200 msec. After the induction of GA, the PHT dropped significantly in Group S compared to Group P. The slight reduction in PHT from baseline in Group P could be due to an increase in left atrial reservoir capacity caused by propofol.^19^ The limitation of the PHT measurement is that it is greatly influenced by the HR and net compliance of the cardiac chambers. Moreover, the presence of more than mild mitral regurgitation could reduce the PHT and underestimate the true MS severity.^14^^,^^15^ Hence, we excluded the patients with associated moderate and severe mitral regurgitation.

Valve area is constant at varying degrees of hemodynamic alterations and hence it serves as a most reliable marker of MS severity. The estimated mitral valve area of less than 1.5 cm^2^ is graded as severe MS.^4^ By echocardiography, the mitral valve area can be estimated by planimetry, continuity equation, PHT, and the Proximal Iso velocity Surface Area (PISA) method.^20^ The valve area estimation by planimetry is considered gold standard but, both planimetry and PISA could not be performed uniformly in all patients due to poor alignment and sub-optimal image in supine lying patients which could have resulted in erroneous measurements. Moreover, the dense calcification of the mitral leaflets underestimates the mitral valve area measurement by the planimetry method.^21^^,^^22^ Hence, we estimated the mitral valve area by PHT and continuity equation for all the study participants in both groups. The significant drop in post-induction PHT in Group S increased the post-induction Mitral Valve Area estimated by PHT (MVA-p). Also, the post-induction MVA-c increased significantly in Group S. In comparison with propofol, the post-induction MVA-c and MVA-p were significantly higher after sevoflurane induction. These observations agree with the findings of the study by Kuperstein et al.^23^ The exact reason for the increase in valve area under anesthesia is not completely understood. It could be the complex interplay between the HR, loading conditions of the heart, myocardial contractility, and interindividual responses to anesthetic agents.^12^^,^^13^ However, the observed increase in MVA-p and MVA-c after induction does not reflect a true anatomical change in valve area but rather the effect of altered loading conditions and HR.

As far as hemodynamic parameters are concerned, both sevoflurane and propofol are known to reduce mean arterial pressure.^13^ In the present study, MAP and cardiac output decreased after induction of GA in both groups. However, these variables were measured repeatedly over time and our analysis relied on separate pairwise t tests rather than a repeated-measures approach like repeated measures ANOVA or a mixed effects model that would better account for within subject correlation and formally assess time-group interaction. Hence, p-values, especially those near the significance threshold, should be interpreted cautiously, as multiple comparisons across time points and outcomes increase the risk of Type I error. Therefore, findings are best considered time-point specific. The underlying mechanism for the hemodynamic change differs slightly between the two anesthetic agents. Reduced venous return and fall in systemic vascular resistance reduce the MAP with sevoflurane induction, whereas the added myocardial depression reduces the MAP with propofol induction.^13^ Theoretically, reduced MAP should augment the CO. Since MS is a fixed output condition, the cardiac output may fail to increase with the fall in MAP. Sevoflurane, a regularly used induction anesthetic agent, is not well studied for its cardio-dynamic effects. However, the available literature supports the reduced response of sevoflurane to vasopressors during induction.^24^ In our study, sevoflurane induction reduced the HR significantly in comparison to propofol. Both sevoflurane and propofol depress the baroreceptor reflex and the net change in HR depends on the balance between the sympathetic and para-sympathetic tone.^25^^,^^26^ As a reflex phenomenon, heart rate should increase with a fall in the MAP. Sevoflurane blunts the baroreceptors more often than propofol to produce a significant reduction in HR.^23^^,^^27^ Also, previous studies have shown that sevoflurane anesthesia resulted in a more stable fall in MAP with a low normal HR.^28^ In MS patients, lower HR is more favorable during the induction of GA.

Heart rhythm and respirophasic changes are the other important influencing factors in assessing the Doppler parameters.^29^^,^^30^ The echocardiographic Doppler parameters vary between the patients in sinus rhythm and those patients with atrial fibrillation due to the abolition of the 'atrial kick' required for adequate left ventricular filling.^31^ Additionally, as part of the induction procedure, the patients were ventilated with positive pressure after administration of the neuromuscular blocking drug, which again could have potentially altered the Doppler parameters. We overcame these two confounding factors by averaging the Doppler measurement over several cardiac cycles as per the ASE guideline recommendations.^11^ It could have nullified the changes in heart rhythm as well as the respiratory cycle which could have otherwise occurred.

We acknowledge that our study has a few limitations. On the outset, we restricted our study to a single ethnic group with two commonly used anesthetic induction agents. Probably, a multinational trial involving a diverse ethnic population group with additional anesthetic agents will be superior in generalizing study results. Next, ACC/AHA recommends the estimation of valve area by planimetry and Pulmonary Artery Systolic Pressure (PASP) for the classification of MS severity.^4^ We could not obtain satisfactory images uniformly to perform the planimetry mitral valve area measurement and not all patients had tricuspid regurgitation for measuring PASP. Also, we did not study the effect of these two agents on postoperative outcomes like duration of mechanical ventilation, length of intensive care unit stay, hospital stay, and mortality. The possible influence of concomitant anesthetic medications and fluid/vasopressors is undeniable. Finally, we could have performed subgroup analysis in those patients with atrial fibrillation. Despite these limitations, our study highlights the short-term hemodynamic effects of sevoflurane as an induction agent in patients with mitral stenosis, suggesting potential areas for further clinical studies.

## Conclusion

In conclusion, both inhalational and intravenous anesthetic induction agents were associated with reductions in trans-mitral pressure gradients following induction of general anesthesia in patients with mitral stenosis. Sevoflurane was associated with more favorable short-term hemodynamic and echocardiographic changes, including an increase in estimated valve area and a reduction in heart rate at the studied time points. As these findings are based on surrogate outcomes from a single center study, further multicenter studies incorporating clinical outcomes are required to establish their clinical significance.

## AI disclosure

The authors did not use generative artificial intelligence in the preparation of this manuscript.

## Data availability statement

The datasets generated and/or analyzed during the current study are available from the corresponding author upon reasonable request.

## Institutional ethics committee approval

(SCT/IEC/1509/JAN-2023)

## Clinical trial registration number

Clinical trials registry of India (Registration Number: CTRI/2023/08/056224; Date: 07, August 2023).

## Funding

None.

## Conflicts of interest

The authors declare no conflicts of interest.
